# Candidate Essential Genes in *Burkholderia cenocepacia* J2315 Identified by Genome-Wide TraDIS

**DOI:** 10.3389/fmicb.2016.01288

**Published:** 2016-08-22

**Authors:** Yee-Chin Wong, Moataz Abd El Ghany, Raeece Naeem, Kok-Wei Lee, Yung-Chie Tan, Arnab Pain, Sheila Nathan

**Affiliations:** ^1^Faculty of Science and Technology, School of Biosciences and Biotechnology, Universiti Kebangsaan MalaysiaBangi, Malaysia; ^2^Chemical and Life Sciences and Engineering Division, King Abdullah University of Science and TechnologyThuwal, Saudi Arabia; ^3^The Westmead Institute for Medical Research and The Marie Bashir Institute for Infectious Diseases and Biosecurity, The University of Sydney, SydneyNSW, Australia; ^4^Codon Genomics SBSelangor, Malaysia

**Keywords:** *Burkholderia cenocepacia*, essential genes, transposon, mutagenesis, TraDIS

## Abstract

*Burkholderia cenocepacia* infection often leads to fatal cepacia syndrome in cystic fibrosis patients. However, antibiotic therapy rarely results in complete eradication of the pathogen due to its intrinsic resistance to many clinically available antibiotics. Recent attention has turned to the identification of essential genes as the proteins encoded by these genes may serve as potential targets for development of novel antimicrobials. In this study, we utilized TraDIS (Transposon Directed Insertion-site Sequencing) as a genome-wide screening tool to facilitate the identification of *B. cenocepacia* genes essential for its growth and viability. A transposon mutant pool consisting of approximately 500,000 mutants was successfully constructed, with more than 400,000 unique transposon insertion sites identified by computational analysis of TraDIS datasets. The saturated library allowed for the identification of 383 genes that were predicted to be essential in *B. cenocepacia*. We extended the application of TraDIS to identify conditionally essential genes required for *in vitro* growth and revealed an additional repertoire of 439 genes to be crucial for *B. cenocepacia* growth under nutrient-depleted conditions. The library of *B. cenocepacia* mutants can subsequently be subjected to various biologically related conditions to facilitate the discovery of genes involved in niche adaptation as well as pathogenicity and virulence.

## Introduction

*Burkholderia cenocepacia*, a member of the *Burkholderia cepacia* complex (Bcc), is an opportunistic pathogen in cystic fibrosis (CF) patients where infection is often associated with deterioration of pulmonary function, resulting in a fatal necrotizing pneumonia known as cepacia syndrome (Mahenthiralingam et al., 2005). *Pseudomonas aeruginosa* and *Staphylococcus aureus* are other dominant CF-associated pathogens and a Bcc infection is implicated in 3–4% of CF patients (McClean and Callaghan, 2009; Cystic Fibrosis Foundation, 2012). Members of the Bcc complex are intrinsically resistant to many antimicrobials with limited treatment choices that rarely result in complete eradication of the pathogen particularly for chronic infections (Leitão et al., 2010; Sousa et al., 2011). Effective antibiotics therapy protocols to combat Bcc infections are currently not well-established, especially toward *B. cenocepacia*, the most resistant Bcc clinical isolate (Leitão et al., 2008; Gautam et al., 2015). Transmission of *B. cenocepacia* is often associated with contaminated hospital water as well as surfaces of medical devices, leading to nosocomial outbreaks (Nasser et al., 2004; Lee et al., 2013). It is unclear how this infectious bacterium gains its ability to adapt to the changing state of nutrient availability. Many strategies have been undertaken to identify virulence determinants that contribute to Bcc pathogenicity (Loutet and Valvano, 2010; Sousa et al., 2011). Nevertheless, target-based drug discovery to combat Bcc infections still remains a major challenge in the absence of knowledge on the molecular mechanisms that contribute to the pathogen’s capacity to adapt and survive within a broad range of environments.

Whole genome sequencing of *B. cenocepacia* strain J2315 revealed that this member of the epidemic ET12 lineage harbors a large 8.06-Mb multi-replicon genome consisting of three circular chromosomes and a plasmid, encoding 7,261 predicted open reading frames in total (Holden et al., 2009). Chromosome 1 harbors a higher proportion of genes encoding for core cellular functions, whereas chromosomes 2 and 3 contain mostly genes encoding for accessory functions. Given that a bacterial genome may undergo genomic reduction or expansion as an indirect consequence of adaptation to variable environmental conditions (Aujoulat et al., 2012), this relatively large genome size raises the question of which genes are indispensable and which are required for Bcc survival and persistence in different niches.

The study of a minimal genome holds the key to understanding fundamental cellular processes. Essential genes encode functions that are absolutely vital for cell growth and viability and this makes these essential proteins an attractive target for the development of novel antimicrobials (Murima et al., 2014). Recently, the discovery of essential genes on a genome-wide basis has become possible because of the advances in DNA sequencing. To date, several tools have been developed to facilitate the screening of essential genes, for example TraDIS (Langridge et al., 2009), Tn-seq (van Opijnen et al., 2009) and INSeq (Goodman et al., 2011), all of which utilize transposon mutagenesis and high-throughput sequencing to assay every gene function on a genome-wide scale. These approaches have successfully identified essential genes of many pathogens, for example *Salmonella enterica* Typhi (Langridge et al., 2009), *B. thailandensis* (Baugh et al., 2013), *B. pseudomallei* (Moule et al., 2014), *P. aeruginosa* (Turner et al., 2015) and *Mycobacterium marinum* (Weerdenburg et al., 2015), demonstrating the robustness of these high-throughput methods in the assessment of gene essentiality.

Here, we describe the construction of a large-scale *B. cenocepacia* J2315 transposon mutant library and the identification of *B. cenocepacia* candidate “essential” genes using TraDIS. High density transposon saturation obtained in our mutant library allowed us to precisely identify a repertoire of candidate genes, most likely indispensable for growth in rich, undefined medium as well as an additional list of condition-specific genes predicted to be important for bacterial growth under nutrient-depleted conditions. A comparison of essential genes identified by TraDIS with that predicted using bioinformatics strategies also provided an overview of what proteins are required to sustain cellular life. This has facilitated a better understanding of *B. cenocepacia* in relation to survival and adaptation to ecological niches whilst proposing potential targets for the development of new antimicrobials.

## Materials and Methods

### Bacteria Strains and Culture Conditions

*Burkholderia cenocepacia* strain J2315 (obtained from the BCCM/LMG Bacteria Collection, Belgium) was used for the construction of transposon mutant pool. The wild type (WT) strain was routinely grown on Ashdown agar and Brain Heart Infusion (BHI) broth at 37°C with agitation (250 rpm). For preparation of electrocompentent cells and recovery of mutants, 2X LB agar supplemented with 500 μg/mL tetracycline were used. *B. cenocepacia* H111 WT strain and three H111 Tn*5* transposon mutants: *lon*::Tn*5, trpA*::Tn*5*, and *trpF*::Tn*5* (Schwager et al., 2013) were used for *in vitro* growth analysis. The mutants were maintained on LB agar supplemented with 50 μg/mL kanamycin.

### Construction of Large-Scale Transposon Mutants

To construct the transposon mutants, the EZ-Tn*5* <TET-1> Insertion Kit (Cat. No. EZ1921T, Epicentre, Illumina) was used. Transposome was prepared according to the manufacturer’s instructions and kept in -20°C until use. Electrocompetent cells were prepared as described by Dubarry et al. (2010) with minor modifications. The overnight bacteria culture was diluted (1:100) in 50 mL SOB medium supplemented with 0.8% glycine until an OD_595 nm_ of 0.08 was reached. The culture was chilled on ice for 30 min and centrifuged at 4°C at for 10 min at 6,000 ×*g*. Harvested cells were then washed three times with pre-chilled 10% glycerol and reconstituted in 200 μL of 10% ice-cold glycerol. Electrocompetent cells were immediately used for electroporation. One μL of transposome was added to 50 μL of freshly prepared electrocompetent cells and the mixture was left on ice for 30 min. The cell suspension was transferred to an ice-cold electroporation cuvette (Cat. No. 1652082, Bio-Rad; 2-mm-wide gap) and pulsed using the Gene Pulser (Cat. No. 1652660, Bio-Rad) at 2.5 kV, 25 μF, and 200 Ω. Immediately after electroporation, 950 μL of SOC medium was added and electroporated cells were incubated at 37°C with agitation for 2 h before being spread on 2X LB agar supplemented with 500 μg/mL of tetracycline, followed by incubation at 37°C for 24 h. The number of mutants obtained was counted and colonies were then washed with BHI broth, scraped off from plates and kept in 30% glycerol at -80°C. Electrotransformations were performed in multiple batches until the number of ∼500,000 mutants was reached. Given that *B. cenocepacia* has a genome size of ∼8 Mb, 500,000 insertions (on the assumption of each mutant is unique) would be sufficient to saturate the genome with one insertion expected every 16 bp. Multiple pools of mutants were then combined to generate a large-scale transposon mutant library or input pool. This input pool was aliquoted and kept in -80°C.

### Screening of Transposon Mutants in Nutrient-Contrasting Growth Media

To prevent any low representation of a particular mutant population within the approximately 500,000 individual mutants in the input pool, each individual mutant was represented by at least 20,000 CFU in the initial inoculum. Approximately 1 × 10^10^ mutants from the input pool were inoculated into 50 mL fresh Luria-Bertani (LB) broth and grown at 37°C with aeration for 24 h (T_1_). Subsequently, 50 μL of this culture (T_1_) was inoculated into 50 mL of fresh LB media and grown for a further 24 h (T_2_) followed by a second passage (T_3_). For M9 minimal medium (supplemented with 0.4% of glucose as carbon source), mutants were grown for 72 h at 37°C (T_1_) and 1 mL of this culture was transferred to fresh medium and grown for a further 72 h (T_2_) (schematic diagram is shown in **Supplementary Materials and Methods Figure S2**). For each output pool, 5 mL of bacteria culture was used for genomic DNA extraction.

### Preparation and Sequencing of TraDIS Libraries

Genomic DNA of the input pool (in duplicate, each representing the same initial library: Input A and Input B) and output pools was extracted using the MasterPure DNA Purification Kit (Cat. No. MCD85201, Epicentre, Illumina). TraDIS libraries were prepared as described by Langridge et al. (2009) with modifications. Generally, 10 μg of genomic DNA was sheared to 100–1500 bp fragments with a sonicator. The fragmented DNA was end repaired and A-tailed with the NEBNext DNA library preparation kit (Cat. No. E6040S, NEB). Adapters were prepared by annealing two oligos, MP_Ad_a and MP_Ad_b. The adapter was ligated to the A-tailed DNA fragments and 200 ng of the adapter-ligated DNA fragments was PCR amplified over 22 cycles using custom made primers Tra_Fp and Tra_Mp_Rp. PCR amplified libraries were then size selected to between 200 and 500 bp in 1% agarose and purified with a Qiagen gel extraction kit (cat #28704). No specific modifications to the above protocol were made to accommodate the high GC content of the *B. cenocepacia* genome. Each DNA template in the sequencing libraries consisted of (1) a 3′ transposon end and its adjacent genomic sequence, (2) Illumina-specific sequences for flow cell binding, and (3) a 7-bp barcode for sample multiplexing (Meyer and Kircher, 2010). Libraries were then quantified using both Bioanalyzer and quantitative real time PCR (qPCR) using primers qPCR_P5 and qPCR_P7. Prepped libraries were sequenced on the Illumina HiSeq 2000 platform with the custom sequencing primer Tra_SeqP and index primer Tra_IndP. Sequences of oligos and primers used in this study and PCR cycling conditions are provided in **Supplementary Materials and Methods**.

### Growth Curve Analysis

Colonies of *B. cenocepacia* H111 WT and Tn5 mutant strains (*lon, trpA*, and *trpF*; Schwager et al., 2013) were inoculated into LB broth and cultures incubated at 37°C for 16 h. Overnight cultures were adjusted to an OD_595 nm_ of 0.5 in LB or M9 minimal medium and 500 μL was transferred to 50 mL fresh media (LB or M9 minimal) and incubated at 37°C with shaking at 250 rpm. At each time point (every 2 h interval), aliquots of bacterial cultures were serially diluted to enumerate the number of live bacteria. For each dilution, 10 μL was dropped onto LB agar (in triplicate) and incubated for 24 h at 37°C. Bacterial CFU for each strain at different time points were then calculated.

### Bioinformatics and Statistical Analysis

Sequence reads from the Illumina FASTQ files were filtered for 10 bases matching the 3′ end of the transposon (TAAGAGACAG) with one mismatch allowed. The transposon tag was removed from the matching reads and the reads were then mapped to the Bcc J2315 reference sequence (EMBL accession numbers AM747720, AM747721, AM747722, and AM747723) using SMALT-0.7.2. The precise transposon insertion site (TIS) was determined using Bio::Tradis^1^. Gene essentiality was assessed as previously described (Langridge et al., 2009). Briefly, an insertion index (number of insertion sites divided by gene length) was calculated for each gene. Insertion indexes were observed to fit a bimodal distribution corresponding to two essential and non-essential sets, each of the modes was modeled using a gamma distribution or an exponential distribution to fit genes with no observed insertion sites. Log_2_ likelihood ratios (LLR) were calculated between the essential and non-essential models and a gene was classified as essential if it had an LLR of < -2. Similarly, a gene was assigned as non-essential if it had an LLR of >2.

The search against available essential genes available in the database of essential genes (DEG;^2^ version 13.3, updated on January 7, 2016; Luo et al., 2014) was performed using BLASTP with the default parameters provided in DEG. BLAST similarities at protein-protein level that resulted in E-values of 10^-10^ or less, were considered matches. *B. pseudomallei* and *B. thailandensis* orthologs of *B. cenocepacia* genes were obtained from the *Burkholderia* Genome Database^3^ (Winsor et al., 2008). For growth curves, doubling time (g) was calculated from the exponential phase using the formula: g = t log 2/(log N_t_ – log N_0_) where N_0_ = number of CFU at a point during log phase, N_t_ = number of CFU at a different time point during log phase and t = time interval between N_0_ and N_t_. The data were expressed as mean ± standard error of the mean (SE) from two independent assays. Statistical analyses were performed using the unpaired, two-tailed Student’s *t*-test.

### Nucleotide Sequence Accession Numbers

Sequence reads were deposited in the database of the European Nucleotide Archive with accession number PRJEB13678 and are accessible via http://www.ebi.ac.uk/ena/data/view/PRJEB13678. The sample accession numbers are ERS1124806 (Input_A), ERS1124807 (Input_B), ERS1124795 (Output LB – T_1_), ERS1124796 (Output LB – T_2_), ERS1124797 (Output LB – T_3_), ERS1124801 (Output M9 – T_1_), and ERS1124802 (Output M9 – T_2_).

## Results

### Construction and Sequencing of *B. cenocepacia* J2315 Transposon Mutant Library

Identification of essential genes by TraDIS requires a large-scale transposon mutant library with appropriate saturation density. To construct transposon insertion mutants, we utilized a transposon mutagenesis system that involves the electroporation of transposome (Tn*5* transposon-transposase complex) into *B. cenocepacia*. It has been reported that genetic manipulation of *B. cenocepacia* strain J2315 can be laborious due to the difficulty in introducing DNA into the bacteria by both electroporation and conjugal transfer (Dubarry et al., 2010). To enhance the electrocompetency of *B. cenocepacia* J2315, we adopted the electrocompetent cell preparation protocol described by Dubarry et al. (2010) where bacteria were cultured in medium supplemented with glycine and harvested at a lower density. This approach enabled us to increase the transformation efficiency by up to three fold. Each electrotransformation generated approximately 2,000 mutants and more than 200 electrotransformations were performed. Multiple batches of mutants were combined to achieve a large-scale library of ∼500,000 mutants, henceforth referred to as the initial library or “input pool.”

**FIGURE 1:**
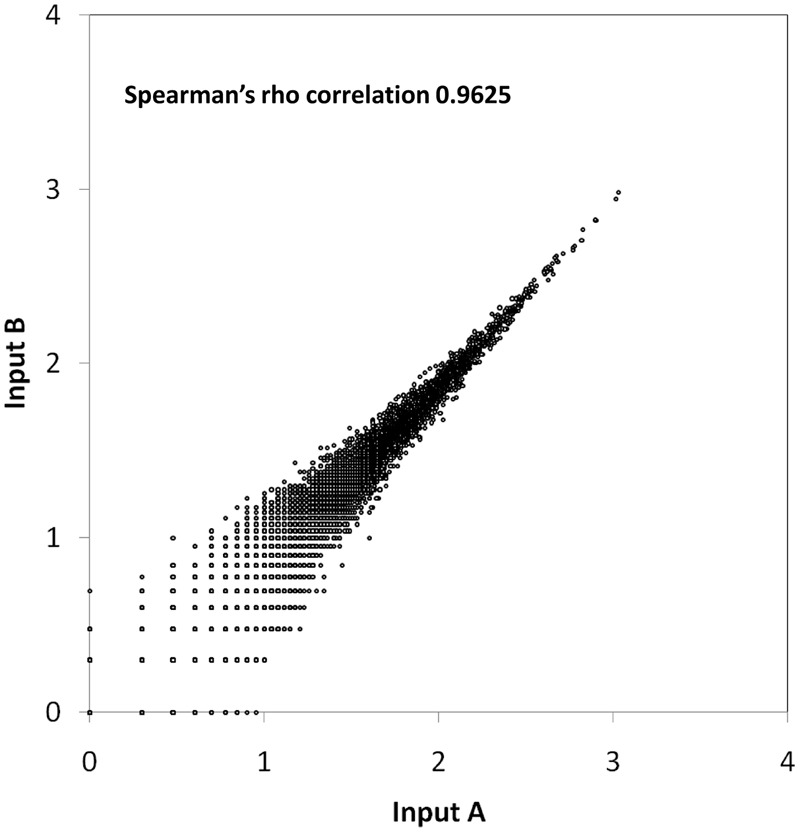
**Scatter plot showing the correlation between two replicates.** The number of unique insertions (log_10_) in each gene from Input A was plotted against Input B. A Spearman’s rho correlation coefficient of 0.9625 indicates low variation between replicates.

Next, we used TraDIS to identify the precise TISs for each mutant (Langridge et al., 2009). A custom sequencing primer was designed to amplify the last 10 bp of the transposon extending into the adjacent bacterial genomic sequence. For each sequencing library, we generated an average of 20 million reads with more than 92% of the total reads containing the 10 bp transposon tag. Non-tagged reads were discarded prior to sequence mapping. Over 82% of the tagged reads obtained from the two technical replicates (henceforth referred to as Input A and Input B) were uniquely mapped to the reference genome resulting in approximately 315,000 and 230,000 unique TIS, respectively, across the genome (**Table 1**). A Spearman’s rho correlation coefficient of 0.9625 indicates low variability in both Input A and Input B, validating the reproducibility of our sequencing data (**Figure 1**). To integrate these two highly correlated data sets, we combined reads generated from both Input A and Input B to increase the sequencing depth and enhance the degree of confidence in identifying unique TIS. In total, we identified 422,585 unique TIS distributed across the genome and the highest density of insertions (about 275,000 unique TIS) was observed in chromosome 1 at an average of one TIS every 14 bp. Approximately 115,000 insertions were mapped to chromosome 2 with one insertion every 27 bp whilst 24,000 TIS were mapped to chromosome 3 with an average insertion gap of 37 bp and about 6,000 TIS were noted on the plasmid at a frequency of one insertion per 16 bp. This high density of insertions obtained was sufficient to saturate the non-essential genes with *Tn5* insertions and also provided the level of genome saturation required to identify essential genes by a negative selection screen.

**Table 1:** Summary of TraDIS results from seven independent sequencing libraries and the respective number of transposon insertions detected in each pool.

Pools	Total reads	No. of reads with transposon tags (%)	No. of reads mapped to the reference genome (%)	No. of unique insertion sites
**Input pools**				
Input A	18,119,795	16,810,854 (92.8)	13,836,384 (82.3)	315,270
Input B	29,297,990	27,098,836 (92.5)	22,277,753 (82.2)	234,601
**Output pools**				
LB – T_1_	27,571,335	26,239,596 (95.2)	16,750,616 (63.8)	405,297
LB – T_2_	28,290,317	27,116,152 (95.9)	20,074,737 (74.0)	336,325
LB – T_3_	25,822,254	24,640,940 (95.4)	18,059,117 (73.3)	394,279
M9 – T_1_	35,969,824	34,790,947 (96.7)	27,146,743 (78.0)	415,180
M9 – T_2_	20,866,529	20,143,720 (96.5)	15,515,399 (77.0)	158,242

### Identification of the Essential Genome of *Burkholderia cenocepacia* J2315

Disruption of a gene crucial for viability can be detrimental and the loss-of-function mutant is non-viable and most probably not present in the initial mutant library. Therefore, we expect to observe a lower or lack of representation of transposon insertions in regions that contain essential genes. In other words, genes with no or very few transposon insertions are most likely to be essential under normal growth or screening conditions. To discount non-disruptive insertions in the 5′ and 3′ ends, we only analyzed TIS located within the sequence spanning the 5–90% portion of each locus as some essential genes may tolerate disruptions at the extreme 3′ and 5′ ends of the open reading frames (Christen et al., 2011; Le Breton et al., 2015). The insertion index of each gene was calculated by normalizing the number of TIS detected per gene by its respective gene length (5–90%). These insertion indexes were then fitted into a bimodal distribution, generating two peaks corresponding to essential and non-essential gene sets (**Supplementary Materials and Methods Figure S1**). A log2-likelihood ratio of less than -2 was assumed to determine the essentiality cutoff. Whilst the LLR ratio may be dependent on the depth of sequencing coverage, a gene with an LLR of < -2 (insertion index value is less than the cutoff value) is at least four times more likely to be essential. It should be noted that the existence of paralogous genes may hamper the interpretation of genome-scale mutagenesis experiments, hence considerable care is needed to discriminate essential from non-essential functions (Peterson and Fraser, 2001). In this study, we expected that there would be no reads mapped to genes with multiple copies or to repeat regions because sequence reads mapped to more than one region were discarded prior to analysis of essentiality. A lack of reads would in turn be translated as “no insertion” leading to these genes being most likely misinterpreted as “essential” by our analysis criteria. An example of this is a 57-kb duplication located in chromosome 1 previously described by Holden et al. (2009), which corresponds to 57 genes (BCAL0969 to BCAL1026 and BCAL2901 to BCAL2846). To prevent this misinterpretation, a total of 231 genes for which reads mapped to these genes can also be aligned to other positions (non-unique) were excluded from the essential list, many of which (40%) were mobile elements mainly insertion sequences (ISs) and putative transposases. By utilizing these stringent selection criteria, we obtained a final list of 383 genes predicted to be essential in *B. cenocepacia* (**Supplementary Data Table S1**). These “essential genes” are defined as the set of genes required to form colonies on 2X LB agar supplemented with tetracycline.

**FIGURE 2:**
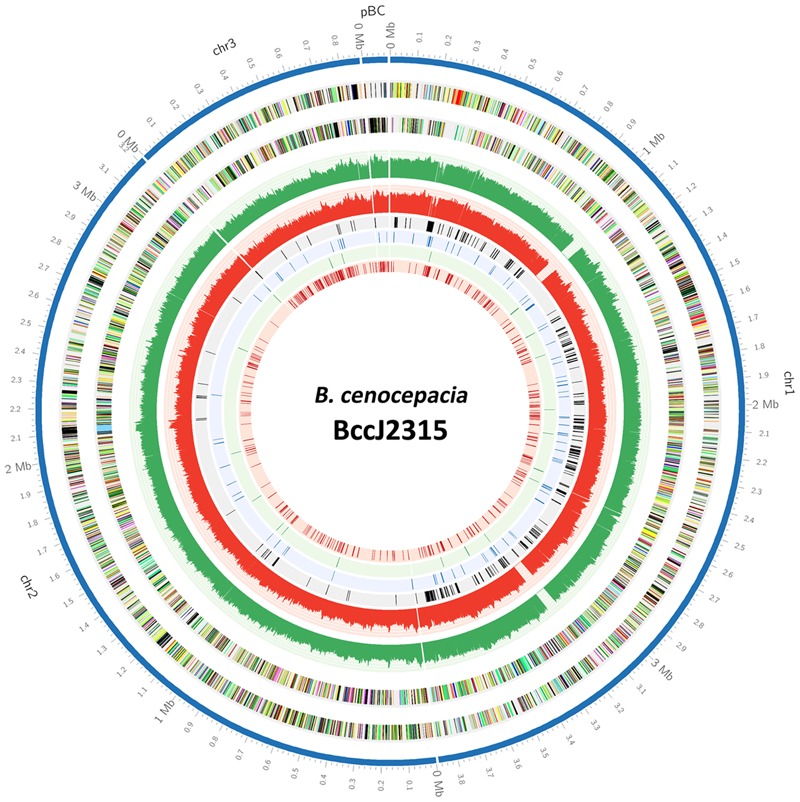
**A Circos-derived (Krzywinski et al., 2009) atlas representation of *Burkholderia cenocepacia* J2315 genome is shown with a base pair (bp) ruler on the outer ring (track 1).** Each chromosome is arranged clockwise, starting with chr1, chr2, chr3, and lastly pBC. The next two outer circles (tracks 2 and 3) represent *B. cenocepacia* J2315 CDS on the forward and reverse strands, respectively. The next two circles (tracks 4 and 5) indicate the number of TIS identified for the input pool over every 1000 bp (green: forward strand; red: reverse strand). The inner four circles present the essential genes predicted for (1) input pool, (2) general *in vitro* growth, (3) LB-specific, and (4) M9 minimal-specific (tracks 6, 7, 8, and 9).

Of these 383 genes, about 90% are located on chromosome 1 (**Figure 2**). Chromosome 1 harbors a higher proportion of genes encoding for core cellular functions (Holden et al., 2009) which explains the biased localization of essential genes to this chromosome. There is a high degree of gene conservation within chromosome 1 of different *Burkholderia* species such as *B. pseudomallei, B. mallei*, and *B. thailandensis* (Lin et al., 2008; Holden et al., 2009), suggesting that the essential housekeeping genes of high functional importance are evolutionarily conserved. Nonetheless, the relative lack of essential genes identified in chromosomes 2 and 3 of *B. cenocepacia* may be due to the different essentiality cutoffs applied to each chromosome to compensate for the uneven distribution of TIS observed (more than 50% of the total TIS found on chromosome 1). As the average gaps between each TIS are found to be larger in other chromosomes, lower essentiality cutoffs limit the number of genes that can be categorized as “essential.”

**FIGURE 3:**
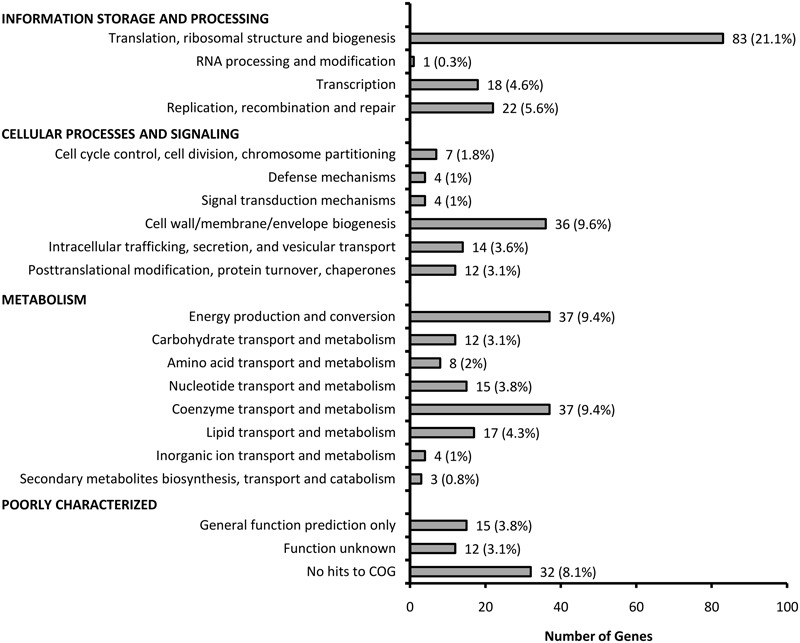
**Distribution of functional categories of the candidate essential genes of *B. cenocepacia* J2315**.

We searched for homologs among genes deposited in the DEGs (Luo et al., 2014) which contains over 15,000 essential genes identified in more than 30 bacterial strains, and we found 87% of our *B. cenocepacia* essential candidates (332/383) had homologs in the DEG. To evaluate the functions encoded by the candidate 383 essential genes, the genes were functionally classified based on the COG (Clusters of Orthologous Groups) annotation system. As depicted in **Figure 3**, the distribution of essential genes in each functional category is typical of that reported for different bacteria (Luo et al., 2014) as well as the synthetic *Mycoplasma mycoides* JCVI-syn3.0, which has a minimal genome smaller than any free-living cell found in nature (Hutchison et al., 2016). About 30% of the genes encode proteins required for information storage and processing, including proteins involved in replication, transcription and translation such as DNA polymerase subunits (*dnaN, dnaQ, dnaX*, BCAL1963, *dnaE*, BCAL2675, and BCAL3371), DNA gyrase subunits (*gyrA* and *gyrB*) and ribosomal proteins (BCAL0222-BCAL0259). Similarly, a large subset of genes (34%) was enriched in metabolic and biosynthesis pathways, for example genes encoding ATP synthase subunits (BCAL0330–BCAL0336) and NADH dehydrogenase subunits (BCAL2331–BCAL2344) required for energy production. About 15% of the genes are poorly characterized with no assigned COG function of which many are annotated as hypothetical proteins. The majority of these genes are also present in other Bcc species and do not have homologs in the DEG suggesting that the essentiality of these genes may be specific to Bcc.

### Conservation of the Predicted Essential Genes among the *Burkholderia* Core Genome

**FIGURE 4:**
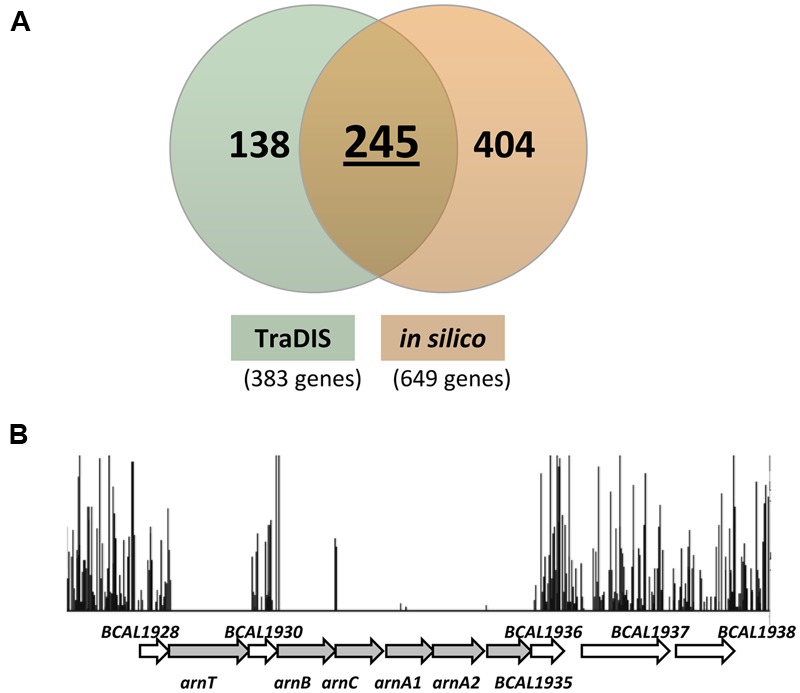
**Comparison of TraDIS predicted essential genes with a previous *in silico*-based prediction. (A)** 383 essential genes (input pool) identified by TraDIS were compared to the list of 649 essential genes predicted using a bioinformatics approach (454 genes homologous to the DEG database and 195 genes without clear orthologs in the DEG database). A total of 245 genes were proposed as essential by both computational and experimental methods; this overlap group is assumed to consist of an accurate representation of *B. cenocepacia* essential genes. **(B)** Insertion profiles of the *arn* gene cluster taken from the initial input pool. Each vertical line represents one unique TIS with the height corresponding to the sequencing depth of the unique read. Genes *arnT, arnB, arnC, arnA1, arnA2*, and BCAL1935 (represented by shaded arrows) are predicted as essential in our TraDIS analysis unlike the *in silico*-derived prediction.

Using a bioinformatics approach, Juhas et al. (2012) presented the core genome of the order *Burkholderiales* consisting of 610 orthologous groups present across 51 *Burkholderiales* species. These groups correspond to 649 genes likely to be indispensable for survival of *B. cenocepacia* in its natural environment. Of these candidates, 454 genes have homologs in the DEG. We extended our analysis to compare our sets of essential genes identified by TraDIS with the list of genes predicted by the *in silico* approach (Juhas et al., 2012). From the total of 383 TraDIS predicted genes, 245 were denoted as essential by computational prediction (**Figure 4A**), including those for core cellular functions such as replication, transcription, and translation as well as cell wall biosynthesis. Suffice to say, this overlapping group consists of an accurate representation of *B. cenocepacia* essential genes, although the number constitutes only about 64% of our TraDIS data set. The discrepancy between experimental and computational prediction of essential genes is most likely due to: (1) the stringent analysis parameters applied to our TraDIS data set limited the number of genes which were categorized as essential; (2) species-specific essential genes may not be represented in the core genome of *Burkholderiales*.

As shown in **Figure 4B**, *arnT, arnB, arnC, arnA1, arnA2*, and BCAL1935 located in the *arn* gene cluster harbor significantly low frequencies of transposon insertions and therefore were categorized as essential in our TraDIS analysis. None of the genes in this cluster was deemed essential by computational prediction. In *B. cenocepacia*, the *arn* gene cluster consists of two transcriptional units encoding enzymes for the biosynthesis of aminoarabinose (Ara4N), a molecule involved in lipopolysaccharide (LPS) modification exploited by Gram negative bacteria to adapt to various stressors (Ortega et al., 2007; Hamad et al., 2012). The addition of Ara4N to lipid A is not required for *in vitro* growth as demonstrated in other bacteria (Kawasaki et al., 2005; Lewenza et al., 2005; Lin et al., 2014) or at least under the laboratory conditions in which previous experiments were conducted. However, it has been shown that disruption of Ara4N biosynthesis in *B. cenocepacia* results in improper assembly of the outer membrane and confers a lethal phenotype (Ortega et al., 2007). Conditional lethal mutants of *arnT* and *arnB* but not the BCAL1928 mutant, exhibited severe growth defects when grown under a non-permissive condition (Ortega et al., 2007). Our TraDIS data supports the essentiality of these genes, as transposon insertions are detectable in the coding region of BCAL1928 but not in *arnT* and *arnB*, confirming that *arnT* and *arnB* are essential. In addition, homologs of *arnT* (BPSL1474), *arnB* (BPSL1472), and *arnC* (BPSL1474) were deemed essential in *B. pseudomallei* K96243 by TraDIS prediction (Moule et al., 2014). Similarly, in *B. thailandensis*, the *arn* gene cluster (BTH_I2189–BTH_I2195) was predicted as essential by Tn-seq (Baugh et al., 2013).

To support our TraDIS prediction, we searched the literature for *B. cenocepacia* rhamnose-dependent conditional mutants that are non-viable when grown in glucose (non-permissive condition). Construction of a conditional lethal mutant generally involves the insertion of a rhamnose-inducible promoter upstream of the target gene, which allows transcription to occur only in the presence of rhamnose. If the target gene is essential, the mutant will not grow in the absence of rhamnose. A study by Bloodworth et al. (2013) reported the identification of *B. cenocepacia* essential operons using conditional growth mutants constructed by rhamnose-inducible promoter replacement of essential genes. About 200,000 *B. cenocepacia* K56-2 transposon mutants were screened for rhamnose-dependent growth and ∼100 mutants showed less than 35% of WT growth in the absence of rhamnose. By determining the insertion sites of the rhamnose-inducible promoter, which controls the expression of downstream genes in the same operon, 50 unique putative essential operons containing 179 genes were identified. Of these, we found 35 operons that contain at least one TraDIS predicted essential gene and together, this corresponds to a total of 77 genes. As expected, these genes encode enzymes that are involved in core metabolic functions such as energy production (ATP synthase subunits and NADH dehydrogenase subunits) and DNA replication (DNA polymerases). A majority of the remaining essential operons (12/15) that do not contain any TraDIS predicted essential genes are small operons which are made up of not more than two genes. Despite these small operons, none of the genes in operon BCAL2388 - BCAL2395, including *purD* which encodes a phosphoribosylamine-glycine ligase and *hemF* which encodes a coproporphyrinogen III oxidase, were deemed essential by our TraDIS prediction. Nonetheless, a total of six genes in this operon have homologs in DEG, suggesting that this operon may be indeed essential in *B. cenocepacia*. In addition, a number of essential genes (*dxs, hemE, infB, gyrB, ubiB, valS, BCAL3369*, and *murJ*) previously validated by a conditional lethal phenotype were also deemed to be essential based on our TraDIS prediction (Cardona et al., 2006; Ortega et al., 2007; Juhas et al., 2012; Mohamed and Valvano, 2014). Hence, we are confident that the TraDIS-based prediction of *B. cenocepacia* essential genes in this study is highly accurate and reliable.

### Identification of Conditionally Essential Genes Associated with *In vitro* Growth

The essentiality of a gene is also dependent on the specific environment that the bacteria is subjected to Peterson and Fraser (2001). Some genes are crucial for the bacteria to adapt to and survive in the environment it occupies where disruption of those genes would result in reduced growth rates or complete growth arrest in that particular environment. Given that *B. cenocepacia* is highly versatile and can thrive in diverse environmental niches, to understand how *B. cenocepacia* adapts to nutrient scarcity, we selected nutrient-depleted medium as a test condition in addition to nutrient-rich Luria-Bertani (LB), a common laboratory medium used in many studies on essential genes. To delineate the subset of genes conditionally essential or critical for growth, we cultured the input mutant pool in nutrient-rich Luria-Bertani (LB) and nutrient-depleted M9 minimal medium. Mutants were grown in LB or M9 over several passages and at each time point, individual output pools were harvested (three time points T_1_, T_2_, T_3_ for LB; two time points T_1_ and T_2_ for M9 as described in the Section “Materials and Methods”). All output pools were sequenced and a summary of the sequencing results is shown in **Table 1**.

**FIGURE 5:**
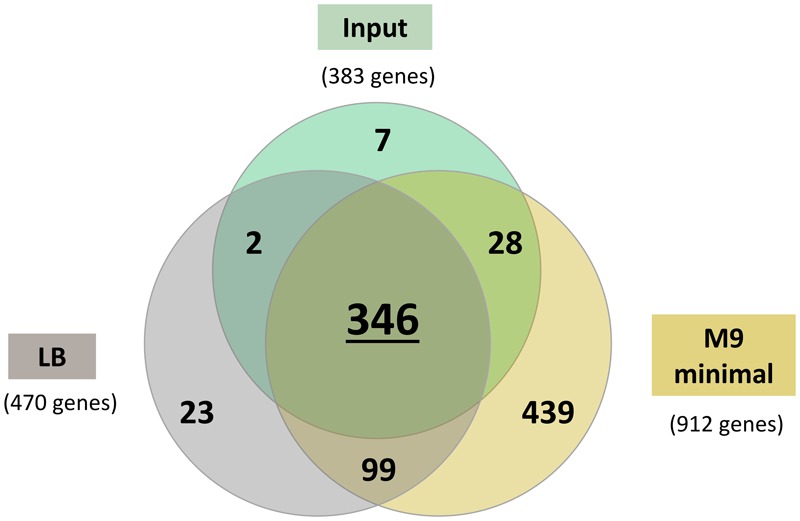
**Venn diagram presenting the number of genes classified as essential in (1) initial input pool, (2) nutrient-rich LB media, and (3) nutrient-depleted M9 minimal media.** A total of 346 genes were essential under all conditions and 99 genes were deemed essential under both LB and M9 minimal and as such were categorized as “general essential genes” required for *in vitro* growth. An additional 439 genes were conditionally essential for bacterial growth in M9 minimal medium.

For every output pool, we assessed the essentiality of each gene using an approach similar to that used for the input pool (**Supplementary Data Table S2**). In the context of this study, genes categorized as conditionally essential/critical for *in vitro* growth are those of which insertions resulted in reduced mutant growth, rather than complete growth arrest as defined for the input pool. To determine the consensus sets of genes critical for growth in LB and M9, we integrated the lists of predicted essential genes for each time point using the following criteria: (1) genes found essential in at least two of the three LB pools; (2) genes found essential in either one of the two M9 pools. This resulted in 470 genes in the LB data set and 912 genes in the M9 data set (**Supplementary Data Tables S3** and **S4**) whereby the number of genes is inclusive of the essential genes predicted for the input pool. Three different data sets (initial input pool, LB and M9) were then compared as shown in **Figure 5**. An overlap of 346 genes were deemed as essential for all conditions and a total of 99 genes were conditionally essential under both LB and M9 growth conditions and we refer to these 99 genes as “general essential” critical for bacterial growth *in vitro*. Up to 463 genes are required for growth under either one of the conditions tested, which we classify as “condition-specific” (**Supplementary Data Table S5**). Genomic locations of the “general, LB-specific and M9-specific” essential genes are shown in **Figure 2**.

**FIGURE 6:**
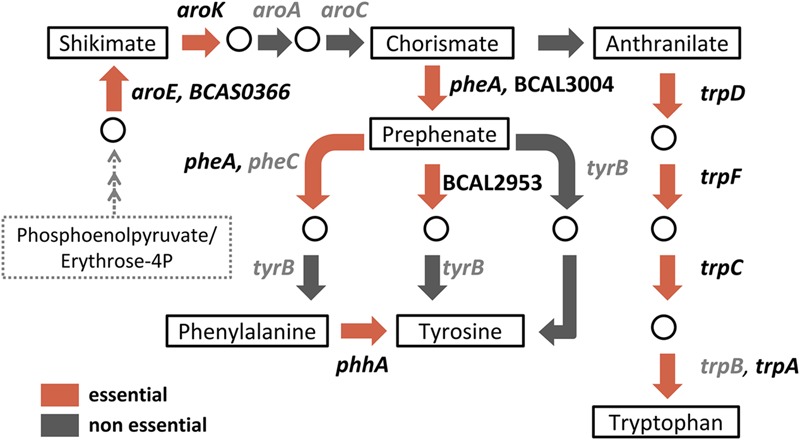
**Schematic diagram of KEGG-derived phenylalanine, tyrosine and tryptophan biosynthesis pathways.** Boxes indicate key products/intermediates. Briefly, over seven metabolic steps, the shikimate pathway converts phosphoenolpyruvate and erythrose 4-phosphate to shikimate which is then converted to chorismate, the precursor of aromatic amino acids. Red arrows indicate reactions associated with predicted conditionally essential genes in M9 minimal medium while gray arrows indicate reactions in which the associated genes are non-essential. Associated genes are listed next to their corresponding reactions: essential genes in black font; non-essential genes in gray font.

A relatively high number of amino acid transport and metabolism genes (71/439) were deemed essential specifically in M9 minimal medium. This observation is consistent with conditionally essential genes identified in other bacteria when grown under nutrient-depleted conditions (Joyce et al., 2006; Lee et al., 2015; Turner et al., 2015). We mapped these genes to KEGG (Kyoto Encyclopedia of Genes and Genomes) pathways and found 34 genes involved in the biosynthesis of amino acids (**Table 2**). Examples of amino acid biosynthesis pathway-related genes identified from our data set are those involved in the biosynthesis of the aromatic amino acids (schematic shown in **Figure 6**). Shikimate pathway converts phosphoenol pyruvate and erythrose 4-phosphate to chorismate, a principal precursor of aromatic amino acids, over seven metabolic steps (Herrmann and Weaver, 1999). *aroK, aroE*, and BCAS0366 which are predicted as conditionally essential genes in our TraDIS analysis, encode components of this pathway. Similarly, deemed conditionally essential are genes involved in the conversion of chorismate to phenylalanine and tyrosine (*pheA*, BCAL2953, and BCAL3004) as well as tryptophan (*trpA, trpC, trpD*, and *trpF*). We suspect that *trp* mutants may be tryptophan auxotrophs and are unable to grow in a tryptophan-depleted environment. It had been demonstrated in *Escherichia coli* that Δ*trpA*, Δ*trpC*, and Δ*trpD* strains could not grow on M9-glucose (Patrick et al., 2009). In *B. cenocepacia* strain H111, *trpA* and *trpB* mutants are auxotrophic for tryptophan and supplementation of the growth medium with tryptophan restored bacterial growth (Schwager et al., 2013) suggesting that *de novo* biosynthesis and transport of amino acids is universally important for bacteria to survive when environmental sources are insufficient.

**Table 2:** List of 34 M9 minimal-specific essential genes involved in biosynthesis of amino acids.

KEGG pathway	Essential genes
Alanine, aspartate, and glutamate metabolism	*glt2*
Arginine and proline metabolism	*BCAL3292*
Arginine biosynthesis	*argB, argH, argG, BCAL2231*
Glycine, serine, and threonine metabolism	*gpmA, BCAL1852, serC*
Histidine metabolism	*hisG, hisD, hisB, hisF, hisI, hisE, BCAL1874*
Lysine biosynthesis	*dapF, BCAS0211*
Phenylalanine, tyrosine, and tryptophan biosynthesis	*phhA, aroK, trpC, trpD, BCAL2953, pheA, BCAL3004, aroE, trpF, trpA, BCAS0366*
Valine, leucine, and isoleucine biosynthesis	*ilvA, ilvI, leuD1, leuB, BCAS0025*

**FIGURE 7:**
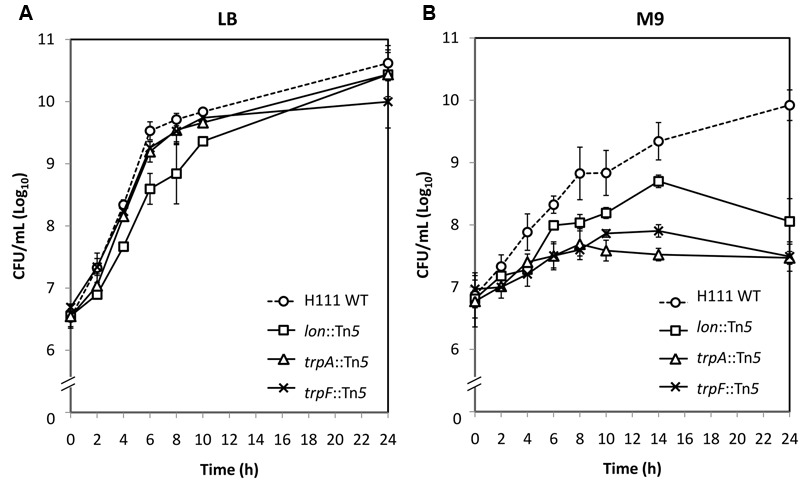
***In vitro* growth of *B. cenocepacia* H111 wild type (WT) and mutants *lon*::Tn*5, trpA*::Tn*5*, and *trpF*::Tn*5* in **(A)** LB and **(B)** M9 minimal media.** When compared to WT, no difference in growth rate was observed for all mutants when grown in LB. When grown in M9 minimal media, all three mutants have slower growth rates when compared to the WT strain. The graph shows the mean ± standard error of the mean (SE) from two independent assays.

As proof of principle, we evaluated the ability of three *B. cenocepacia* H111 Tn*5* mutants (*trpA*::Tn*5, trpF*::Tn*5*, and *lon*::Tn*5*) to grow in both liquid LB and M9 minimal media as compared to the WT isogenic H111 strain. As the *trpA, trpF*, and *lon* mutants were lost from the TraDIS output pools as a result of multiple passages in liquid media, we postulated that disruption of *trpA, trpF*, and *lon* do not lead to bacterial cell death, but rather, result in reduced growth rates when the individual mutants are grown in either LB or M9 medium. *trpA* (BCAM0993) and *trpF* (BCAM0990), which encode tryptophan synthase alpha chain and *N*-(5′-phosphoribosyl)anthranilate-isomerase respectively, are predicted to be M9-specific, whilst *lon* (BCAL1994), which encodes an ATP-dependent protease, is conditionally essential for bacterial *in vitro* growth in both liquid LB and M9 medium. As shown in **Figure 7A**, growth rates of all three mutants in LB are comparable to that of the WT strain, with doubling times of 0.76 ± 0.04 h (WT), 1.01 ± 0.29 h (*lon*::Tn*5*), 0.69 ± 0.06 h (*trpA*::Tn*5*), and 0.85 ± 0.14 h (*trpF*::Tn*5*). At early exponential phase, although not significant (*p* > 0.05), *lon*::Tn*5* mutant showed a slightly slower growth rate. When subjected to M9 minimal medium, *trpA*::Tn*5* and *trpF*::Tn*5* mutants exhibited significantly slower growth (doubling times of 7.10 ± 0.19 h and 4.16 ± 0.81 h respectively) as compared to the WT (doubling time of 1.54 ± 0.10 h; *p* < 0.05; **Figure 7B**), thereby validating our TraDIS prediction. This observation is also consistent with previous findings that *trpA*::Tn*5* and *trpF*::Tn*5* mutants could not grow in ABC minimal medium (Schwager et al., 2013). Taken together, our results confirm that *trpA* and *trpF* are essential for *B. cenocepacia* growth in nutrient-depleted medium but not in enriched medium. In addition, the *lon*::Tn*5* mutant showed a much slower growth rate (doubling time of 2.47 ± 0.04 h) when grown in M9 minimal, suggesting that *lon* is in fact required for *B. cenocepacia* growth under nutrient limiting condition. Although the *lon*::Tn*5* mutant was predicted to grow slower in liquid LB, we did not observe any significant decrease in the overall growth rate. As the TraDIS screen generally involves a mixed bacterial population and is a competition assay by itself, it is possible that the outcome of the TraDIS prediction may not be similarly reflected in this single-strain experiment. The disruption of *lon* may not directly lead to significant growth reduction in LB medium, rather, it affects the overall competitive fitness of the resulting mutant in a mixed population.

## Discussion

A growing interest in defining the minimum number of genes necessary to sustain cellular life has led to extensive studies on determining the repertoire of genes indispensable for bacterial growth and viability under various environmental conditions. With the increasing number of complete genome sequences currently available, a comprehensive search for essential genes in a genome-wide manner has become more feasible. As highly conserved genes shared between organisms most likely encode for essential functions, *in silico* comparative genomic analysis between phylogenetically distant species has been used to define essential genes (Peterson and Fraser, 2001; Jordan et al., 2002; Gil et al., 2004). For instance, a comparative analysis of the *B. pseudomallei* K96243 genome sequence with essential genes of other bacteria deposited in the DEG resulted in the prediction of 312 essential proteins (Chong et al., 2006). Similarly in *B. cenocepacia*, identification of genes conserved across 51 *Burkholderia* species revealed the core genome of the order *Burkholderiales* consisting of 649 genes, many of which are predicted to be essential (Juhas et al., 2012). However, a major drawback of this bioinformatics-based approach is the lack of experimental evidence and in many cases, predicting gene function by sequence homology is limited to existing databases and also prone to uncertainties (Ponting, 2001; Lee et al., 2007). Saturation transposon mutagenesis coupled with next-generation sequencing is a robust experimental method for high-throughput identification of essential genes. TraDIS was first described in *S. enterica* Typhi, in which parallel sequencing of 1 million transposon mutants enabled a simultaneous assay of gene function in a genome-wide manner (Langridge et al., 2009). This robust technique also facilitated the discovery of 505 essential genes in *B. pseudomallei* (Moule et al., 2014) and 406 genes in *B. thailandensis* using a similar transposon sequencing approach, Tn-seq (Baugh et al., 2013). This prompted us to employ TraDIS as an experimental approach to facilitate the identification of *B. cenocepacia* essential genes. A combination of computational and experimental methods to determine essential genes may contribute to a better understanding of the basic functions required to sustain cellular life (Juhas et al., 2011).

A signature-tagged mutagenesis (STM) study on *B. cenocepacia* to identify genes required for bacterial survival *in vivo* described the construction of 2,627 individual tagged transposon mutants (Hunt et al., 2004). Schwager et al. (2013) screened 5,500 independent transposon mutants using a *Caenorhabditis elegans* infection model to identify *B. cenocepacia* H111 virulence factors and a study on H111 biofilm regulation reported the construction of 40,000 individual mutants (Aguilar et al., 2014). In the effort of identifying essential genes, more than 200,000 *B. cenocepacia* K56-2 mutants constructed by random replacement of rhamnose-inducible promoter were screened for rhamnose-dependent growth (Bloodworth et al., 2013). Construction of insertional knock-out mutants by electroporating preformed transposase–transposon complexes (transposomes) into the cells offers a simple and efficient way to achieve a large-scale transposon mutant library (Goryshin et al., 2000). In this study we used Tn5 transposome to successfully construct a library of approximately 500,000 *B. cenocepacia* J2315 transposon mutants. This pool of mutants represents the largest number of *B. cenocepacia* insertional knockout mutants described to date.

Massive parallel sequencing of this saturated pool of transposon mutants using TraDIS facilitates genome-wide mapping and quantification of precise insertion sites. TIS were distributed across the genome (**Figure 2**) confirming transposon saturation in our mutant pool. In total, we achieved over 400,000 independent insertion sites, with the highest density marked on chromosome 1 (i.e., one insertion in every 14 bp). This is 60% higher than that reported in *B. pseudomallei* (∼240,000 insertions identified for 1 million mutants; Moule et al., 2014) and *B. thailandensis* (∼200,000 insertions identified for 200,000 mutants; Baugh et al., 2013). In addition, we noticed a higher density of insertions in chromosome 1, a similar observation previously noted for *B. pseudomallei* (Moule et al., 2014) which suggests insertional bias and the potential to underestimate the essential genes in chromosomes 2 and 3. To overcome this issue, we applied a higher level of stringency toward our analysis on the other chromosomes compared to chromosome 1 to compensate for the differences in insertion density. As *B. cenocepacia* has one of the largest genomes known among Gram-negative bacteria comprising of multiple chromosomes, we believe that this is by far the largest bacterial genome investigated using TraDIS and other approaches such as Tn-seq.

Assessment of gene essentiality by negative selection allowed us to identify a total of 383 *B. cenocepacia* J2315 essential genes with a vast majority of these genes concentrated in chromosome 1. These genes are mostly involved in core cellular processes and pathways such as DNA replication, protein synthesis, energy metabolism as well as cell wall biosynthesis, similar to those reported in other bacteria (Phan et al., 2013; Moule et al., 2014; Le Breton et al., 2015). About 10% of the essential genes are located in chromosomes 2 and 3. As reported by Holden et al. (2009), these two components contain essential genes and are designated as chromosomes rather than megaplasmids. However, Agnoli et al. (2012) demonstrated that the loss of chromosome 3 does not affect *B. cenocepacia* H111 growth under standard laboratory conditions (non-essential) and should be reclassified as a megaplasmid (pC3). Based on our TraDIS-derived data, we identified 12 predicted essential genes on the J2315 chromosome 3, including the elements required for its replication (e.g., *parA* and *parB*) and also genes most likely involved in chromosome 3-dependent metabolic phenotypes such as D-xylose utilization (Agnoli et al., 2012) and fatty acid utilization (Subramoni et al., 2011). A recent study by Du et al. (2016) has shown that loss of the chromosome 3 Par system severely retarded J2315 growth. Nonetheless, it is unlikely that xylose and fatty acid utilization genes are essential. As the number of transposon insertions located on chromosome 3 is relatively low, non-essential genes might be categorized as “essential” as a result of the relatively large gap between each TIS (average ∼40 bp). On the other hand, it is also plausible that both H111 and J2315 pC3 replicons contain distinctive sets of genes and hence, the essentiality of this third replicon for cell viability may also be strain-dependent. Currently, we are unable to conclusively address whether chromosome 3 is absolutely dispensable for J2315 viability and this warrants further investigation. For plasmid pBCJ2315, only *parA* and *parB* are predicted to be essential. However, it has been shown that the deletion of plasmid *parAB* does not affect growth (Du et al., 2016), supporting the dispensability of this plasmid. In the context of this study, it remains not known how plasmid *parAB* is essential for J2315 viability.

**FIGURE 8:**
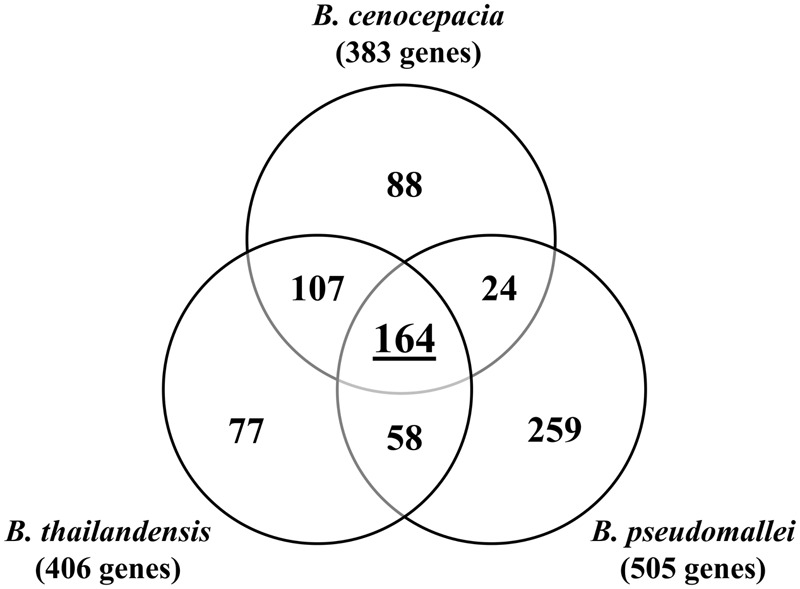
**Essential genes identified in *B. cenocepacia, B. pseudomallei* (Moule et al., 2014) and *B. thailandensis* (Baugh et al., 2013).** A total of 164 genes are found to be essential in all three *Burkholderia* species.

We compared the identified 383 *B. cenocepacia* essential genes to the *B. pseudomallei* and *B. thailandensis* essentials identified previously (Baugh et al., 2013; Moule et al., 2014; **Figure 8**). A total of 382 *B. cenocepacia* genes are orthologs of the 406 *B. thailandensis* essential genes and more than 70% (271/382) of the *B. cenocepacia* orthologs were identified as essential in our study. Of the 505 *B. pseudomallei* essentials, there are 346 *B. cenocepacia* orthologs but only 188 (54%) were deemed essential in our data set. This is possibly due to a relatively low number of essential genes identified in *B. cenocepacia* (383 genes) compared to *B. pseudomallei* (505 genes). The more stringent analysis parameters applied to our TraDIS interpretation most likely limited the number of genes that can be proclaimed as essential particularly genes located in chromosomes 2 and 3. We propose that the genes deemed non-essential in *B. pseudomallei* and *B. thailandensis* are *B. cenocepacia*-specific essentials. Using experimental approaches such as TraDIS and Tn-seq, a total of 164 genes are predicted as essential in all three *Burkholderia* species and this repertoire of genes may represent the core essential genome of *Burkholderia* species (**Figure 8**).

In addition, our TraDIS-derived data also confirmed the important role of the *arn* gene cluster which encodes proteins required for aminoarabinose (Ara4N) biosynthesis and LPS modification for *B. cenocepacia* viability (Ortega et al., 2007). The Ara4N modification of lipid A is a regulated mechanism exploited by many Gram negative bacteria to resist cationic antimicrobial peptide, and under most growth conditions this modification is dispensable (Kawasaki et al., 2005; Lewenza et al., 2005; Loutet and Valvano, 2011; Lin et al., 2014). Hamad et al. (2012) demonstrated that Ara4N served as a recognition signal for the transport of LPS to the outer membrane through the Lpt pathway, consolidating the essential role of this aminoarabinose in determining the architecture of the outer membrane in *B. cenocepacia*. The *arn* gene cluster is also predicted as essential in *B. pseudomallei* and *B. thailandensis* (Baugh et al., 2013; Moule et al., 2014), hence, this pathway represents an appealing target for the development of *Burkholderia*-specific inhibitory molecules.

The ability of bacteria to monitor and respond to nutritional cues enables them to adapt to living in different environments. Our study has also revealed an additional subset of genes conditionally essential for *B. cenocepacia* growth under a nutrient-depleted environment. Studies by Lee et al. (2015) and Turner et al. (2015) described the essential genes required for *P. aeruginosa*, also a significant CF pathogen, to grow in MOPS minimal medium and most interestingly, in CF sputum. In *P. aeruginosa*, a majority of the genes required for growth in minimal medium are involved in the biosynthesis of amino acids, nucleotides and cofactors. By comparing the LB- and M9-derived lists of conditional essential genes, we found several genes involved in the biosynthesis of aromatic amino acids phenylalanine, tyrosine and tryptophan (*trpA, trpD, trpC, pheA, aroE*, and *aroK*) conditionally essential in both *P. aeruginosa* and *B. cenocepacia*. Aromatic amino acids are potential mediators responsible for the increased synthesis of the quorum sensing signal molecule, *Pseudomonas* quinolone signal (PQS), when *P. aeruginosa* is grown in CF sputum (Palmer et al., 2005). *B. cenocepacia trp* mutants have been reported to be less virulent in a nematode infection model and the observed attenuation is possibly due to the decreased production of AidA, a quorum sensing-regulated virulence factor that has been shown to be important in the killing of *C. elegans* (Schwager et al., 2013). Thus, it is conceivable that bacterial aromatic amino acids biosynthetic pathways respond to host environmental signals during infection, which may then trigger quorum sensing-mediated regulation of virulence factors.

Furthermore, genes involved in the synthesis of the cofactor biotin, *bioB, bioD*, and *bioA* which were deemed conditionally essential in *B. cenocepacia*, were also essential for *P. aeruginosa* growth in CF sputum (Turner et al., 2015). Given that the level of biotin readily available in CF sputum may not be sufficient to allow maximal bacterial growth (Turner et al., 2015), it is reasonable that the biosynthesis of biotin becomes crucial for bacterial survival. We propose that *B. cenocepacia* also requires the biosynthesis of biotin to survive in and adapt to CF sputum and further investigation is needed to confirm this hypothesis. Taken together, these biosynthetic pathways may be essential for nutrient acquisition and metabolism of infecting bacteria in the host environment and therefore represent potential targets for drug development (Murima et al., 2014).

## Conclusion

We have demonstrated the successful application of TraDIS to identify *B. cenocepacia* J2315 essential genes. Studies on gene essentiality will contribute to the understanding of fundamental functions needed to support cellular life as well as the discovery of potential targets for the development of new antimicrobials. Furthermore, condition-specific essential genes identified in this study provide a general overview of bacterial adaptation to nutrient limitation and serve as a framework for future studies on how nutritional cues affect Bcc pathogenicity in the host environment. Most importantly, the *B. cenocepacia* transposon mutant pool generated in this study serves as the foundation for further studies, particularly on host-pathogen as well as environment–pathogen interactions.

## Author Contributions

Y-CW, MA, AP, and SN conceived and designed the experiments. Y-CW performed the experiments. RN, K-WL, and Y-CT performed part of the bioinformatics analysis. Y-CW, MA, AP, and SN analyzed the data and wrote the paper.

## Conflict of Interest Statement

The authors declare that the research was conducted in the absence of any commercial or financial relationships that could be construed as a potential conflict of interest.

## Footnotes

1 https://github.com/sanger-pathogens/Bio-Tradis

2 http://tubic.tju.edu.cn/deg/

3 http://www.burkholderia.com

## Supplementary Material

SUPPLEMENTARY DATA **Lists of *B. cenocepacia* J2315 general and conditional-essential genes identified in this study**.

SUPPLEMENTARY MATERIALS AND METHODS **Sequences of oligos and primers used and detailed information of *in vitro* passage**.
