# Supplementary material for: Candidate Essential Genes in Burkholderia cenocepacia J2315 Identified by Genome-Wide TraDIS
Source: Front Microbiol. 2016 Aug 22;7:1288. doi: 10.3389/fmicb.2016.01288 (PMC4993015; doi:10.3389/fmicb.2016.01288)
Supplement: SUPPLEMENTARY MATERIALS AND METHODS — Sequences of oligos and primers used and detailed information of in vitro passage. [file Data_Sheet_2.DOCX]

Supplementary Material

Candidate essential genes in *Burkholderia cenocepacia* J2315 identified by genome-wide TraDIS

**Yee-Chin Wong^1^, Moataz Abd El Ghany^2,3^, Kok-Wei Lee^4^, Yung-Chie Tan^4^, Arnab Pain^2^, Sheila Nathan^1^***

*** Correspondence:** Sheila Nathan: sheila@ukm.edu.my

# Oligo/primers used for TraDIS

| Oligo/primer | Sequence (5' - 3') | Description |
| --- | --- | --- |
| MP_Ad_a | GTGACTGGAGTTCAGACGTGTGCTCTTCCGATC*T | Adapter ^a^ |
| MP_Ad_b | p-GATCGGAAGAGCGTCGTGTAGGGAAAGAGTG-amino |  |
| Tra_Fp | AATGATACGGCGACCACCGAGATCTACACCTGATCTAGAGTCGACCTGCAGGCATGCAAGCTTCAG | Amplification of TraDIS library; Forward and reverse primer^b^ |
| Tra_Mp_Rp | CAAGCAGAAGACGGCATACGAGATNNNNNNNGTGACTGGAGTTCAGACGTGT |  |
| Tra_qPCR_P5 | AATGATACGGCGACCACCGA | TraDIS library quantification by qPCR |
| Tra_qPCR_P7 | CAAGCAGAAGACGGCATACGA |  |
| Tra_SeqP | AGGCATGCAAGCTTCAGGGTTGAGATGTGTA | Sequencing primer |
| Tra_IndP | GATCGGAAGAGCACACGTCTGAACTCCAGTCAC | Index primer^c^ |

^a^Both oligonucleotides were modified from Illumina adapters. Asterisk indicates phosphorodioate bond, p represents 5’ phosphate modifier and amino represents 3’ amino modifier. Underlined is a 12-bp complementary region, both oligonucleotides anneal to form a Y-shaped adapter.

^b^N signifies 7 bp barcodes as described by Meyer and Kircher (2010).

^c^Index primer was designed according to Illumina primer sequences.

Oligonucleotide sequences © 2007-2012 Illumina, Inc. All rights reserved. Derivative works created by Illumina customers are authorized for use with Illumina instruments and products only. All other uses are strictly prohibited.

# Indexes for sample multiplexing

| Sample^a^ | 7-bp barcode^b^ |
| --- | --- |
| Input A | CCTAGGT |
| Input B | GGATCAA |
| Output LB-T_1_ | TCGCAGG |
| Output LB-T_2_ | CTCTGCA |
| Output LB-T_3_ | CCTAGGT |
| Output M9-T_1_ | TCGCAGG |
| Output M9-T_2_ | CTCTGCA |

^a^Samples that were sequenced on the same flow cell lane: (1) Input A and Input B; (2) Output LB-T_1_, Output LB-T_2_, and Output LB-T_3_; 3) Output M9-T_1_ and Output M9-T_2._

^b^as described by Meyer and Kircher (2010).

# TraDIS library preparation: PCR Cycle Conditions

Template: adapter-ligated DNA fragments (200 ng)

Primers: Forward: Tra_Fp; Reverse: Tra_MP_Rp.

Each sample uses reverse primer with different index or barcode (Meyer and Kircher, 2010).

94°C 2 min

94°C 30 sec

65°C 30 sec 22 cycles

72°C 30 sec

72°C 5 min

# Supplementary Figures


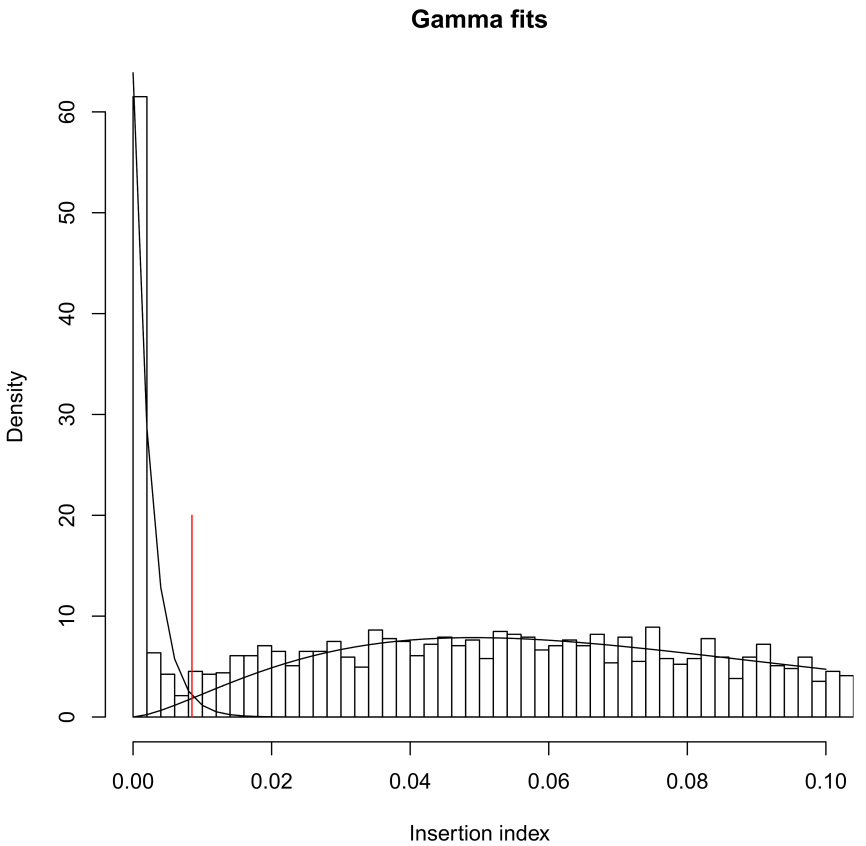


**Figure S1**: A representative density plot showing the bimodal frequency distribution of insertion index (number of inserts per gene divided by gene length). Genes with insertion indices in the leftmost peak represent those that have none or very few insertions (essential genes). The red line shown indicates the essentiality cut-off; genes with insertion indexes lower than the cut-off value are categorized as essential.


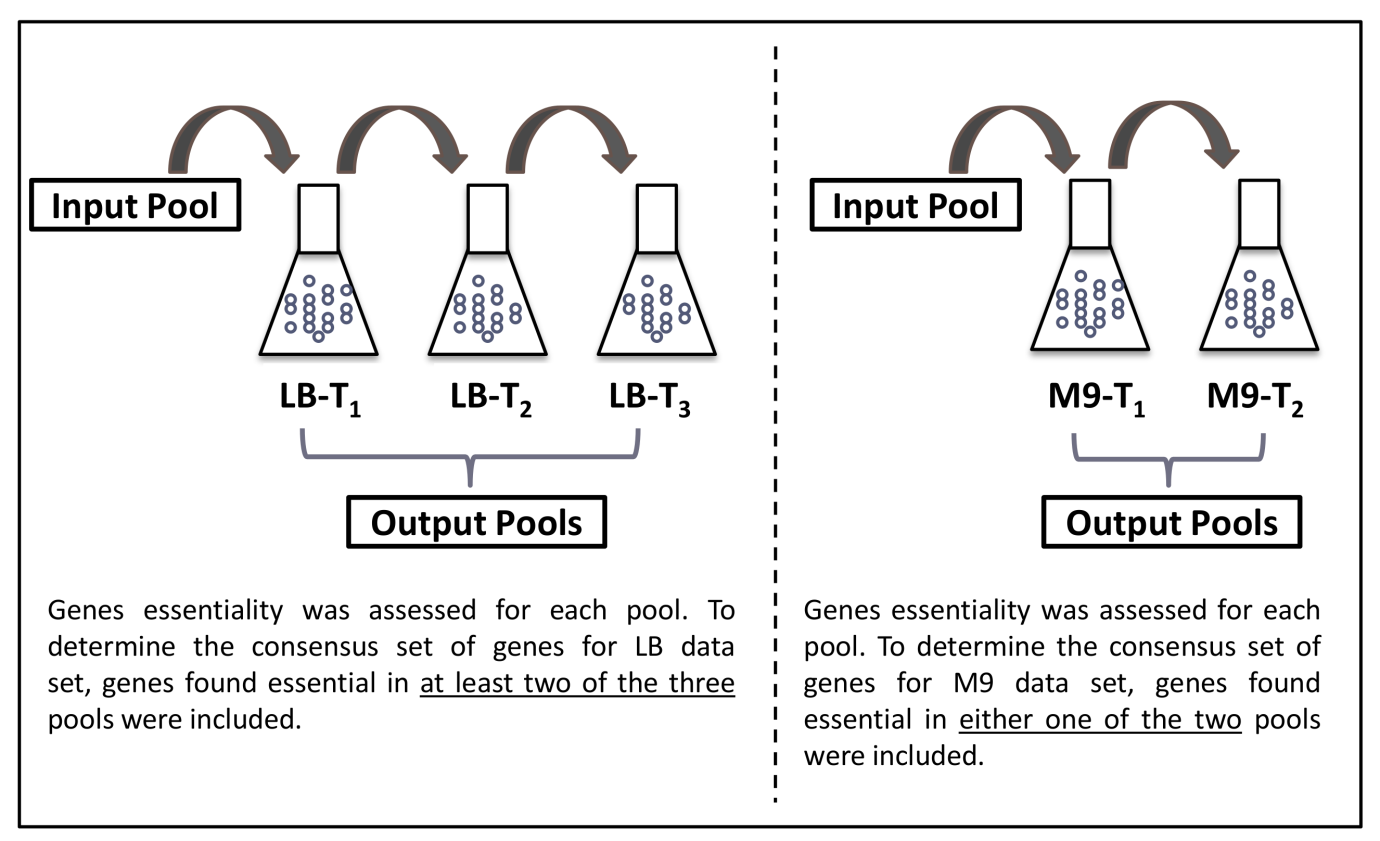


**Figure S2**: Schematic diagram showing mutant *in vitro* passage in LB and M9 minimal media. For LB, mutants were grown for 24 h before transferred to fresh medium. For M9 minimal, mutants were grown for 72 h.
